# Report of the Convalescent Homes Committee

**Published:** 1916-12-23

**Authors:** 


### KING EDWARD'S HOSPITAL FUND—

### REPORT OF THE CONVALESCENT HOMES COMMITTEE.

(1) The Governors and General Council have this year fixed the sum available for distribution amongst convalescent homes and consumption sanatoria at £7,500, as against £6,500 in 1915. This is the largest sum that has ever been placed at the disposal of the Convalescent Homes Committee.

(2) The number of applications eligible for consideration amounted to forty-two from convalescent homes and nine from consumption sanatoria, as against forty-one and nine respectively last year.

VARYING RELATIVE CLAIMS.

(3) In dealing with all applications, whether from sanatoria or from convalescent homes, the Committee have, as usual, taken into consideration the question of comparative urgency of need, as shown by the audited accounts and balance sheets of the various institutions to December 31 last. In the case of consumption sanatoria they have also taken note of the proportion of the total work of each institution which is covered by the receipts under the National Insurance Act. As these and other circumstances affecting the relative claims of the different institutions vary from year to year, it must not be assumed that a reduction in the grant to any particular institution, whether for maintenance or for capital purposes, or even the absence of a grant, implies dissatisfaction.

(4) The proportion allotted to convalescent homes is again smaller than usual owing to the absence of grants to several homes which since the beginning of the war have ceased to be occupied by convalescent patients from London, and are therefore temporarily ineligible for consideration by this Committee.

### KING EDWARD'S HOSPITAL FUND—(*continued*).

More Consumption Sanatoria Beds.

(5) The Committee have been able to make a slight increase this year in the number of beds reserved at consumption sanatoria for the use of patients in London hospitals, owing to the fact that certain women's beds that are no longer available have been replaced by children's beds. The accommodation thus secured this year amounts to fifty-three beds at six sanatoria, situated as follows:

Nine beds for children at the Children's Sanatorium, Holt, Norfolk.

Four beds at the Daneswood Sanatorium (Jewish), Woburn Sands, Beds.

Ten beds at the Devon and Cornwall Sanatorium, South Brent, Devon.

Six beds at the Kelling Sanatorium, Holt, Norfolk.

Twelve beds at the Sanatorium of the National Association for the Establishment and Maintenance of Sanatoria for Workers, Benenden, Kent.

Twelve beds at the Northampton Sanatorium, Creaton, Northants.

(6) The Committee recommend that the beds thus reserved be allocated amongst London hospitals as follows:

Twelve beds to the City of London Hospital for Diseases of the Chest.

Two beds to the Royal Hospital for Diseases of the Chest.

Eleven beds to the London Hospital.

Ten beds to Guy's Hospital.

Five beds to the Middlesex Hospital.

Five beds to St. George's Hospital.

Four beds to St. Mary's Hospital.

Four beds to University College Hospital.

(7) The visitors' reports, often made at the expense of long journeys and considerable inconvenience, have again proved most useful to the Committee, and in suitable cases extracts from the observations of the visitors have been privately attached to the grants.

(8) The grants recommended to the various institutions are appended.

For the Committee,

December 1916. Edwin Freshfield, Chairman.

